# Molecular and epidemiological characterization of imported malaria cases in Chile

**DOI:** 10.1186/s12936-020-03353-y

**Published:** 2020-08-13

**Authors:** Daniel F. Escobar, Naomi W. Lucchi, Rispah Abdallah, María Teresa Valenzuela, Venkatachalam Udhayakumar, María Isabel Jercic, Stella M. Chenet

**Affiliations:** 1 Sección de Parasitología, Instituto de Salud Pública de Chile, Santiago, Región Metropolitana Chile; 2 Malaria Branch, Division of Parasitic Diseases and Malaria, Center for Global Health, Centers for Disease Control and Prevention, Atlanta, GA USA; 3 Facultad de Medicina, Universidad de los Andes, Santiago, Chile; 4 Instituto de Investigación en Ganadería y Biotecnología, Universidad Nacional Toribio Rodríguez de Mendoza, Amazonas, Peru; 5 Instituto de Enfermedades Tropicales, Universidad Nacional Toribio Rodríguez de Mendoza, Amazonas, Peru

**Keywords:** Malaria, Surveillance, Chile, *pfcrt*, *pfmdr1*, Microsatellites, PCR

## Abstract

**Background:**

Chile is one of the South American countries certified as malaria-free since 1945. However, the recent increase of imported malaria cases and the presence of the vector *Anopheles pseudopunctipennis* in previously endemic areas in Chile require an active malaria surveillance programme.

**Methods:**

Specimens from 268 suspected malaria cases—all imported—collected between 2015 and 2018 at the Public Health Institute of Chile (ISP), were diagnosed by microscopy and positive cases were included for epidemiological analysis. A photo-induced electron transfer fluorogenic primer real-time PCR (PET-PCR) was used to confirm the presence of malaria parasites in available blood samples. Sanger sequencing of drug resistance molecular markers (*pfk13*, *pfcrt and pfmdr1*) and microsatellite (MS) analysis were performed in confirmed *Plasmodium falciparum* samples and results were related to origin of infection.

**Results:**

Out of the 268 suspected cases, 65 were *Plasmodium* spp. positive by microscopy. A total of 63% of the malaria patients were male and 37% were female; 43/65 of the patients acquired infections in South American endemic countries. Species confirmation of available blood samples by PET-PCR revealed that 15 samples were positive for *P. falciparum*, 27 for *Plasmodium vivax* and 4 were mixed infections. The *P. falciparum* samples sequenced contained four mutant *pfcrt* genotypes (CVMNT, CVMET, CVIET and SVMNT) and three mutant *pfmdr1* genotypes (Y184F/S1034C/N1042D/D1246Y, Y184F/N1042D/D1246Y and Y184F). MS analysis confirmed that all *P. falciparum* samples presented different haplotypes according to the suspected country of origin. Four patients with *P. vivax* infection returned to the health facilities due to relapses.

**Conclusion:**

The timely detection of polymorphisms associated with drug resistance will contribute to understanding if current drug policies in the country are appropriate for treatment of imported malaria cases and provide information about the most frequent resistant genotypes entering Chile.

## Background

Malaria is a mosquito-borne infectious disease caused by parasites of the genus *Plasmodium*. The four species of *Plasmodium* that commonly infect humans are *Plasmodium falciparum*, *Plasmodium vivax*, *Plasmodium ovale*, and *Plasmodium malariae*. The fifth human malaria species is *Plasmodium knowlesi*, known to cause simian malaria, which has been reported in nearly all countries from Southeast Asia and in travellers visiting these countries [1, 2].

In 2017, there were 219 million estimated cases of malaria worldwide, resulting in 435,000 deaths [3]. In South America, the most prevalent species are *P. vivax* and *P. falciparum,* with 773,000 malaria cases estimated in 2017; 97% of these cases were found in Brazil, Colombia, Peru, and Venezuela [1, 4]. Recently, there has been a significant increase in the number of cases in Venezuela due to the political unrest leading to lack of available treatment [3, 5]. Colombia and Brazil also reported more confirmed cases compared to previous years, which shows an increase of the transmission in the region for this period [3, 6, 7].

Chile has been certified as a malaria-free country by the World Health Organization (WHO), with no indigenously-acquired cases reported since 1945 [8]. This was possible due to the implementation of an anti-malaria campaign in the early 1940s directed towards vector (*Anopheles pseudopunctipennis*) control and the prompt treatment of malaria patients [8]. Between 1945 and 2001, there were 90 imported cases of malaria and five malaria-related deaths reported by the Chilean Ministry of Health [8]. Malaria is a disease of immediate notification according to decree DTO N° 158/04 as part of the Emerging Diseases Surveillance of the Ministry of Health. Samples from all suspected cases are sent to the Public Health Institute of Chile (ISP) for diagnostic confirmation.

Currently, *An. pseudopunctipennis* (known malaria vector) breeding sites have been found in close proximity to residential areas in Valle de Azapa, Valle de Lluta, Quebrada de Camarones and Chaca in the Arica y Parinacota Region and in the Tarapacá Region. Additionally, González and Sallum, reported the presence of a new species of *Anopheles* with unknown vector potential in Atacama Region, northern Chile [9].

For many years, the recommended malaria treatment was chloroquine (CQ) followed by sulfadoxine–pyrimethamine (SP) until resistant parasites spread worldwide. Currently, artemisinin-based combination therapy (ACT) is the first-line treatment recommended by the WHO for *P. falciparum* infected patients in countries where CQ and SP resistance has been reported [10]. However, the treatment for *P. vivax* infections is chloroquine plus primaquine (CQ + PQ), with a variation of 7 or 14 days of therapy with PQ depending on the country [4].

Publications reporting delayed parasite clearance following ACT in Southeast Asia [11–13] placed a worldwide alert on the importance of monitoring malaria drug resistance. In South America, the first *P. falciparum* isolates harbouring the C580Y mutation in *P. falciparum kelch 13* gene were reported in samples collected in 2010 in Guyana [14]. This mutation is highly prevalent in Southeast Asia and is a confirmed marker of artemisinin resistance [12, 15, 16]. Although ACT is still highly effective in many parts of the world, there is a serious concern about the emergence of artemisinin resistance in other parts of the world.

While malaria transmission is not known to occur in Chile, importation of malaria cases from other South American countries and other parts of the world requires proper monitoring in order to inform treatment policy. In this study, a molecular characterization of parasites from imported malaria cases was undertaken to confirm infecting *Plasmodium* species, mixed infections, and to assess the drug resistance profile of parasites. Additionally, microsatellite (MS) markers were analysed in *P. falciparum* samples to explore the presence of clusters of cases and/or polyclonal infections.

## Methods

### Malaria samples

As part of national malaria surveillance, samples from suspected malaria cases throughout Chile are collected from public and private health centers and sent to the Public Health Institute of Chile (ISP) for diagnosis. A database search was performed at ISP to collect epidemiological information of patients with suspected malaria from January 2015 to December 2018 using identifiers such as: age, gender, travel history, malaria treatment, health center and region of origin. This subsequent work was part of public health surveillance and therefore Institutional Review Board (IRB)/consent was not needed, and personal information was excluded for publication.

Malaria infections in these samples at the time of diagnosis were determined by microscopy using Giemsa-stained blood smears performed at the ISP. All malaria positive patients were treated with atovaquone–proguanil and where possible, patients were followed up to ascertain clearance of their infection and were requested to report to the clinic if symptoms returned. Each health centre and treating physicians defended when and how to take additional samples for follow up, since this determination is not within the powers of the ISP.

This descriptive study used data from 268 suspected malaria cases received by the ISP during 2015–2018, period in which 65 positive patients with the *Plasmodium* spp. were confirmed through microscopy using Giemsa-stained blood smears. For molecular analysis, 46 samples were included, which was the total number of available blood samples for subsequent analysis in the laboratory. These 46 samples corresponded to 39 different patients.

### DNA isolation and genotyping methods

Genomic DNA was extracted from 350 µl of blood using the EZ1 DNA blood Kit and the automated system EZ1 Advanced XL (QIAGEN, Valencia, CA) following the manufacturer´s instructions. Samples were eluted in 100 µl and screened using the multiplex photo-induced electron transfer (PET) real-time PCR (PET-PCR) for species confirmation [17, 18]. PET-PCR assays were run with 5 μl of DNA template using Agilent Mx3005pro thermocyclers (Agilent Technologies, Santa Clara, CA, USA). As previously established, a *C*_*T*_ value of < 40 was considered positive; samples with *C*_*T*_ values above 40 were considered to be negative. DNA from confirmed *P. falciparum* samples were subsequently sequenced to examine single-nucleotide polymorphisms (SNPs) in the genes: *P. falciparum* chloroquine resistance transporter (*pfcrt*), *P. falciparum* multidrug resistance associated protein 1 (*pfmdr1*), and *P. falciparum kelch13* (*pfk13*) using protocols from previous studies [14, 19, 20]. Sequencing of PCR products was performed using an ABI 3130 (Applied Biosystems, CA, USA). The sequence data was analysed using the Geneious Pro R8 software (Biomatters Inc, Newark, NJ).

### Microsatellite analysis

Seven *P. falciparum* neutral MS (TA1, Polyα, PfPK2, TA109, C2M34, C3M69, and 2490) located in chromosomes 2, 3, 4, 6, and 12 were amplified using previously published methods [21, 22] since this methodology for *P. falciparum* was already established in the laboratory. Post-PCR fluorescently labeled products were separated on an Applied Biosystems 3130 capillary sequencer and scored using GeneMarker v1.95 (SoftGenetics LLC). The presence of more than one additional allele in a single locus was interpreted as a coinfection with more than one genetically distinct clone in the patient. Missing data (no amplifications) were not considered for defining haplotypes. The size of the alleles obtained in this study was normalized with 1 bp for analysis and comparison with published alleles previously.

## Results

### *Plasmodium* species identified and origin of cases

**Fig. 1:**
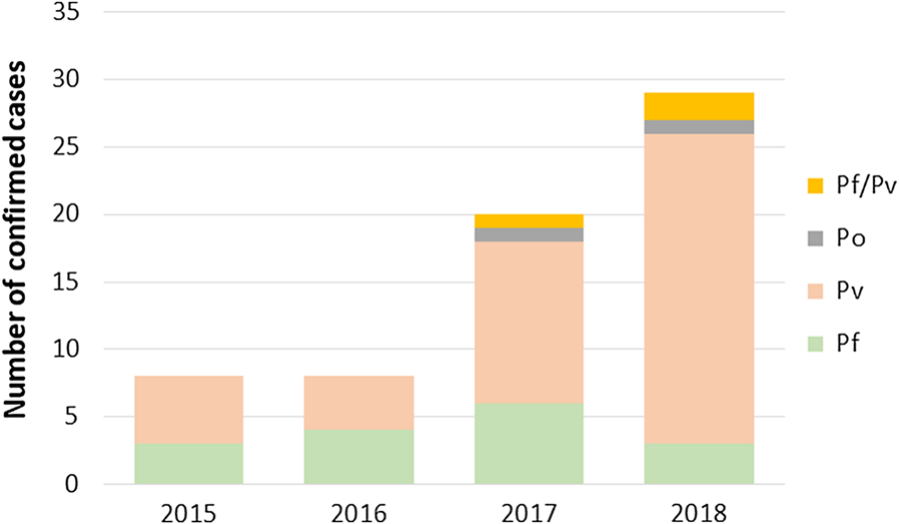
Malaria positive cases confirmed by microscopy between 2015 and 2018

Microscopy analysis at the ISP confirmed that 65 of the 268 suspected cases were positive for malaria (24.3%), 16 (24.6%) were positive for *P. falciparum*, 44 (67.7%) for *P. vivax*, 2 (3.1%) for *P. ovale*, and 3 (4.6%) were mixed infections (*P. falciparum* and *P. vivax*) (Fig. 1). PET-PCR results of the 46 available samples (blood samples from all microscopy positive cases were not available), showed that 15 samples were positive for *P. falciparum*, 27 for *P. vivax,* and 4 were mixed infections (*P. falciparum*/*P. vivax*). Two samples previously identified as *P. vivax* by microscopy were reported as mixed (*P. falciparum*/*P. vivax*) infections by PET-PCR. Follow up samples (microscopy slides) were taken for 12 patients; however; only four presented symptoms and confirmed *P. vivax* infections more than 28 days after the initial infection (Table 1). These patients were treated following the guidelines established by the Chilean Ministry of Health.

**Table 1:** Patients with follow up samples

Patient ID	Day	Gender	Age	Site of infection	Region^a^	Result
3	D0	M	28	Brazil	Metropolitana	*P. vivax*
	D126					*P. vivax*
18	D0	F	42	Colombia	Aysén	*P. vivax*
	D115					*P. vivax*
21	D0	F	20	Peru	Arica y Parinacota	*P. vivax*
	D112					*P. vivax*
27	D0	M	48	Colombia	Tarapacá	*P. vivax*
	D1					*P. vivax*
	D96					*P. vivax*

^a^Location of the Health Centres in Chile. The follow up samples were determined by the treating physicians

**Fig. 2:**
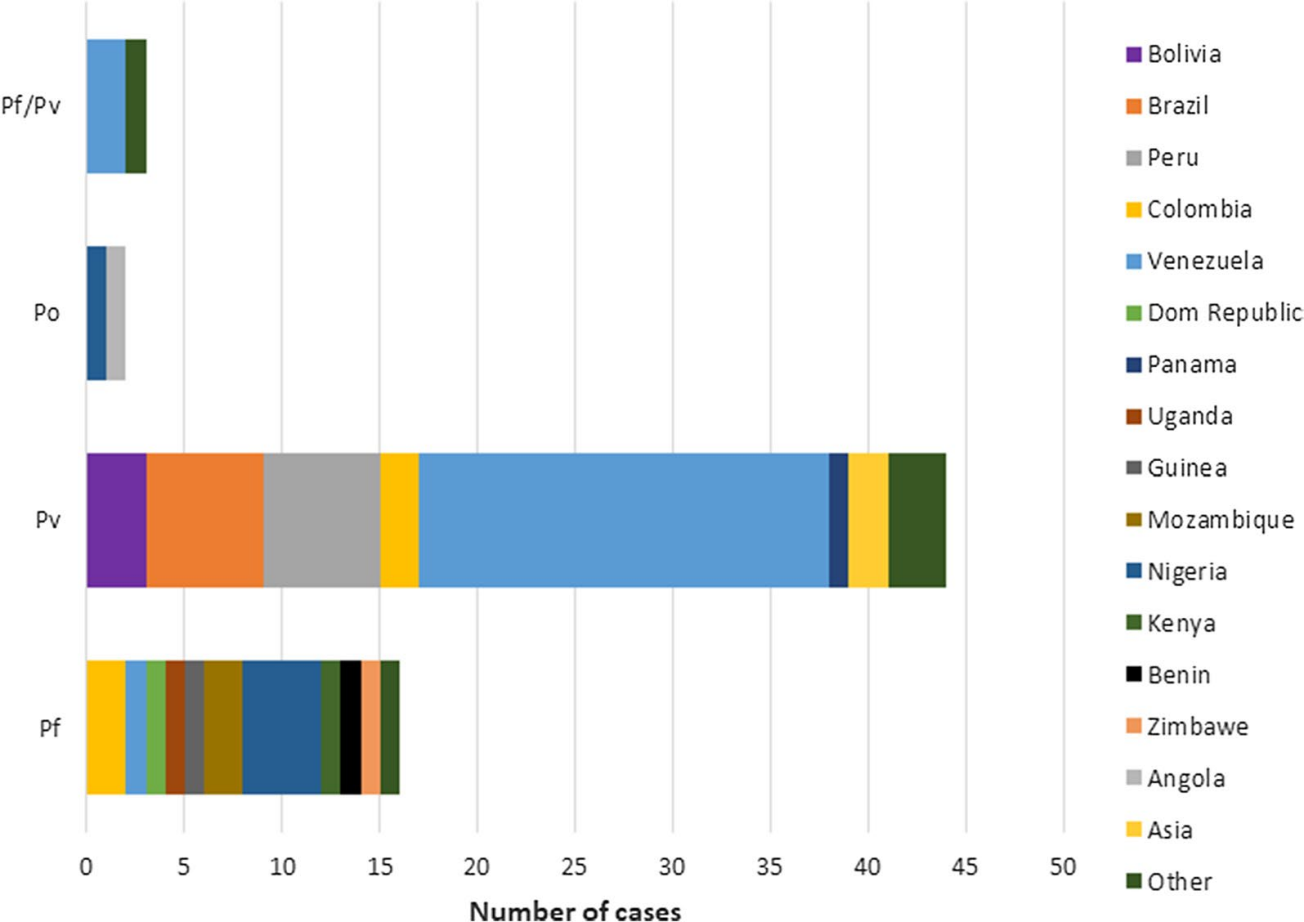
Origin or reported travel history of microscopy confirmed malaria cases collected between January 2015 and December 2018

**Fig. 3:**
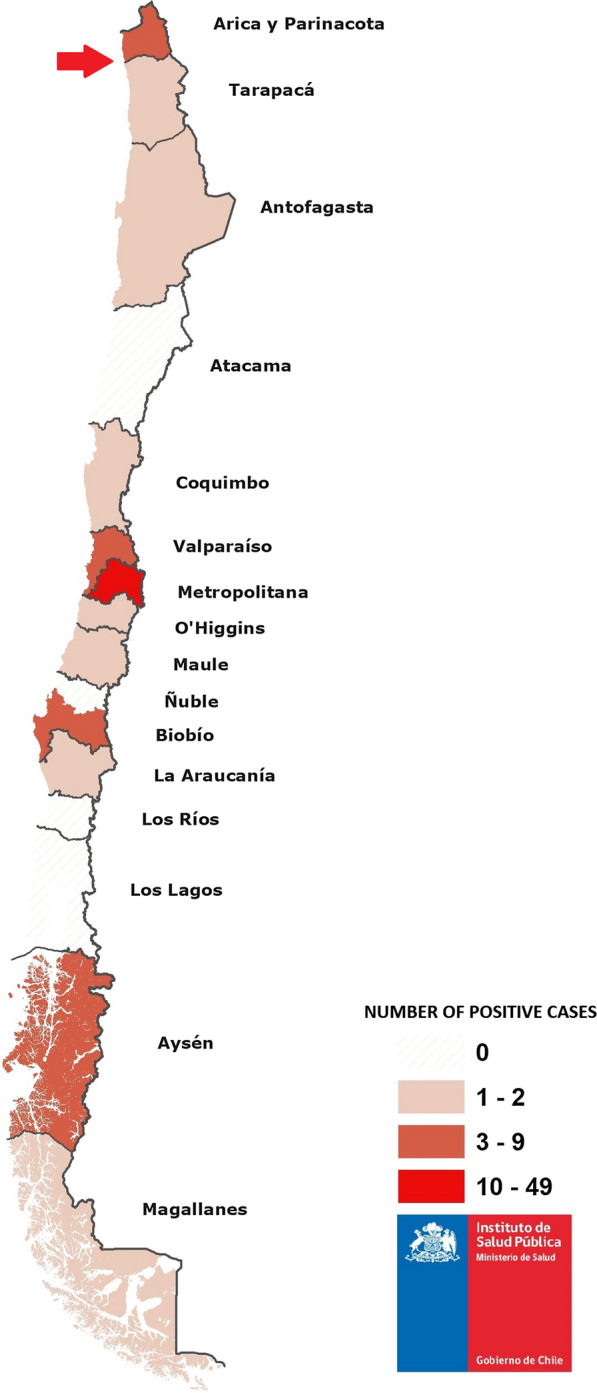
Heat map of regions reporting malaria positive cases from 2015 to 2018 using ArcGIS version 10.2 software. Samples analysed were collected from public and private health centres throughout Chile. The red arrow indicates the presence of *Anopheles* mosquito

According to the epidemiological information of positive cases, 63% were male while 37% were female. The age distribution was: nearly half (47.7%; 31) of patients were 26–40 years old, 3 (4.6%) were 5–14 years old, 11 (16.9%) were 15–25 years old, 10 (15.4%) were 41–50 years old, and 9 (13.8%) were > 50 years old; 1 patient’s age was not reported. According to the travel history, 61.7% of the positive cases came from South American countries, mainly from Brazil, Colombia, Peru, and Venezuela, 24.7% of the patients reported travels to Africa, and 13.6% of the patients traveled to Central America, Asia, and other countries (Fig. 2). Additional information showed that most of the positive samples (60.5%) were collected in health centres located in the Metropolitan Region of Santiago, the capital of Chile and the main port of entry to the country. Importantly, 7.4% of the samples were collected in the Region of Arica and Parinacota and in the Region of Tarapacá, where *Anopheles* breeding sites have been described (Fig. 3).

### Molecular characterization of *Plasmodium falciparum* parasites

Additional genetic analysis for drug resistance markers were undertaken in the *P. falciparum* positive samples, given that drug resistance has been confirmed in this species. From a total of 19 *P. falciparum* samples, 17 were properly amplified and they were available for this analysis. All samples had the wild type *pfk13* genotype, while *pfcrt* showed mutant genotypes with the triple-mutant CV**IET** (codons 74–75–76) (Uganda, Guinea and Benin), double-mutant CVM**ET** (codons 75–76) (Colombia/Nicaragua), double-mutant **S**_tct_VMN**T** (codons 72–76) (Venezuela), and single-mutant CVMN**T** (codon 76) (Colombia) (Table 2). Wild type *pfcrt* genotypes were found in samples from patients with travel history to Mozambique, Kenya, Nigeria, and the Dominican Republic, according to data provided in the clinical file. On the other hand, a quadruple *pfmdr1* mutant Y184**F**/S1034**C**/N1042**D**/D1246**Y** was found in a sample from Venezuela, a triple-mutant Y184**F**/N1042**D**/D1246**Y** in Colombia and a single mutant Y184**F** in Uganda, Nigeria and Dominican Republic. The wild *pfmdr1* genotype was found in people with travels to Guinea, Mozambique, Kenya, and Benin (Table 2).

**Table 2:**
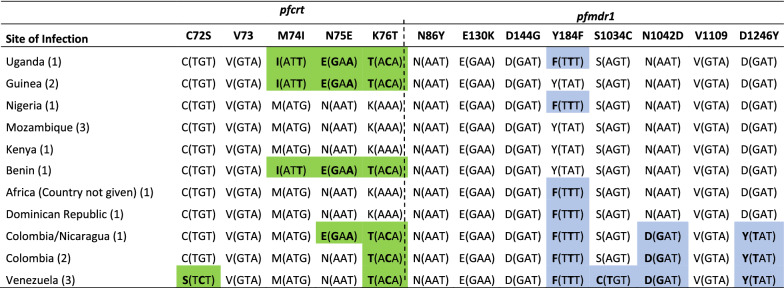
Drug resistance profiles of the *P. falciparum* samples

Nucleotide substitutions and changes in amino acid composition are highlighted in bold and colors: green (*pfcrt*); light blue (*pfmdr1*)

According to the MS analysis, no clustering of cases was found. Most of the samples presented polyclonal infections; however, samples from Venezuela, Dominican Republic, one sample from Nigeria and one from Mozambique showed monoclonal infections (Table 3).

**Table 3:** Microsatellite profiles of the *P. falciparum* samples analysed

Country of origin/travel	TA1 (Ch5)	Polyα (Ch4)	PfPK2 (Ch7)	TA109 (Ch6)	2490 (Ch10)	C2M34 (Ch2)	C3M69 (Ch3)
Uganda (1)	141–183	165	162–171–180	151–163	81	234	138
Guinea (2)	141–171	123	177	178	81	228	124
Nigeria (1)	141	147	174	160	72	238	124
Mozambique (1)	141	135–165–195	168–189	124–133–175–199	78	220	150
Mozambique (1)	141	165	189	175	81	250	150
Mozambique (1)	141–177	135	162	175	96	238	146
Kenya (1)	141–165	156	x	x	81	260	128
Benin (1)	162	159	168–192	187	78-81	250	146
Africa (1)	141	150–153–186	183	160–199	78	230	146
Dominican Republic (1)	174	150	159	175	81	218	124
Colombia/Nicaragua (1)	141–171	147	162	160	78	226	140
Colombia (1)	141–171	147	160	160	78	226	140
Colombia (1)	141–171	156	171	178	81	238	146
Venezuela (2)	141	150	171	163	78	240	134
Venezuela (1)	171	186	164	175	78	240	134

X = no amplification

## Discussion

The increase in the number of suspected and confirmed malaria cases in Chile might be associated with the high movement of Chileans traveling for work and/or pleasure to endemic countries, such as Brazil, Colombia, and Peru. Additionally, according to the National Institute of Statistics of Chile (INE), 746,465 immigrants (particularly from Haiti, Venezuela, Colombia, and Peru) live in Chile and it is estimated that this number has increased more than 300% compared to the previous decade (2010–2017: 471,285 and 2016–2017: 220,090) [23].

Although malaria has been eliminated in Chile, globalization and cultural exchange has contributed to raising awareness of the disease and for that reason early detection of malaria cases have improved in recent years. It is necessary that health care providers in Chile obtain the travel history of patients in order to diagnose malaria promptly, notify health authorities and send samples for confirmation to the ISP. Moreover, malaria should be included in the differential diagnosis for every patient with fever who has travelled to an area where malaria is endemic [24].

In this study, the most frequent malaria cases were due to *P. vivax* infections. Unfortunately, complete travel history, prophylaxis, and anti-malarial treatment could not be obtained for all cases. Nevertheless, as expected, the travel history matched the infecting species expected, with *P. vivax* infection identified in people who visited *P. vivax* endemic countries in South America and Asia and *P. falciparum* mainly from Africa. While no clusters were detected in our analysis, a cluster of *P. vivax* cases was previously reported among Chilean travellers returning from Peru [25]. This highlights the need to promote the use of chemoprophylaxis in travellers, especially, in those visiting specific locations in endemic countries with high malaria transmission.

In Chile, imported malaria cases have been reported in areas that were previously considered endemic and where the presence of the *Anopheles* vector has been confirmed. The existence of imported malaria cases, which are possible reservoirs of the infection, together with the occurrence of favourable climatic conditions for competent vector species, represent a risk for the onset of autochthonous malaria cases in the country. Therefore, vigilance and continued surveillance should be warranted.

Additionally, the drug resistance profiles of the parasites are important to establish the guidelines for an appropriate treatment policy. Mutations in the *pfcrt* gene reported in this study (CVMN**T**, CV**IET**, CVM**ET**, **S**_**tct**_VMN**T**) are known chloroquine resistance markers found worldwide. Their frequency in endemic countries and the possible origin of each genotype is associated with different geographical locations [26, 27]. For instance, the CV**IET** genotype is typically found in Africa, in countries such as Uganda, Guinea and Benin [26, 28–34]. The literature also reports the presence of this genotype in countries such as India, Thailand, Vietnam, Cambodia and Papua New Guinea [27]. In South America, it has been reported in Peru, Brazil and Guyana. The **S**_**tct**_VMN**T** genotype, found in samples from Venezuela, has two possible independent origins, one in South America and one in Papua New Guinea [20, 27]. The CVM**ET** genotype found in a sample from a patient with travel history to Colombia and Nicaragua is mainly reported in the northern part of South America. The CVMN**T** genotype identified in two samples from Colombia is the closest to the ancestral sensitive genotype, which has been previously reported in Brazil, Colombia, Ecuador and Peru. The wild-type *pfcrt* genotype is commonly found in Central America [27, 28, 35].

There were several *pfmdr1* alleles identified in the samples analyzed, showing single, triple, and quadruple mutants. Mutations at *pfmdr1* N86**Y** and D1246**Y**, which are common in Africa, have been linked to decreased sensitivity to chloroquine and amodiaquine, but increased sensitivity to lumefantrine, mefloquine, and artemisinins [36]. Other polymorphisms, primarily seen outside Africa (including 1034**C** and 1042**D**), are associated with altered sensitivity to lumefantrine, mefloquine, and artemisinins [36]. Samples from patients who traveled to Uganda, Nigeria, and the Dominican Republic showed a single mutation in codon 184 (Y184**F**), which has been previously reported in these countries [37–39]. MS analysis of *P. falciparum* confirmed that the samples analyzed were all imported, clusters or clonal expansions were not found. Furthermore, the haplotypes identified in this study, were all different (Table 3). The MS alleles found in the sample from Uganda, were consistent with previous findings of these alleles in this country. The analysis of alleles showed that four of the seven loci belong to haplotypes previously described in Uganda: TA1 (183), Polyα (165), Pfpk2 (162/171/180) and TA109 (163) [40].

In addition, the 2490 allele “81” found in the sample from Uganda has also been found in other African countries such as Kenya [41]. Furthermore, the TA1 (141/165) and Polyα (156) alleles found in the patient returning from Kenya, are commonly found in several localities of this country [41, 42]. Also, the patient’s sample with travel history to Guinea revealed a consistent MS pattern with a previous report from this country [43]. The TA1 allele (171) found in isolates from Guinea and Colombia, has been previously reported in other countries from South and Central America but also in low frequency in Guinea and Mozambique [35, 44–46]. The sample from Nigeria also revealed a consistent pattern with previous reports on loci PfPK2 and TA109 [47]. The haplotypes found in samples from Colombia and Venezuela in this study, also presented alleles previously detected in both countries and in others from South America [44, 45]. The samples from Colombia showed a haplotype profile akin to one previously reported in Colombia, from the town of Nariño [44]. One of the samples from Colombia corresponded to a patient with travel history to Nicaragua. However, the haplotype profile suggests the infection was acquired in Colombia. Finally, the MS profile identified in the sample from the Dominican Republic was similar to the one reported by Carter et al., in the Island of Hispaniola in Central America [48].

An important aspect within an Integrated Surveillance System, such as the one Chile is currently implementing, is to join efforts to obtain complete epidemiological information on all cases reported, including using adequate diagnostic tools in primary health centers, following up all malaria cases, recommending appropriate prophylaxis to Chilean travellers, and ensuring the availability of appropriate drugs for malaria treatment. Currently, atovaquone–proguanil (malarone) or mefloquine could be used as the first-line treatment for patients with uncomplicated malaria in Chile [49]. Among the patients included in this study, four reported post-treatment relapses with incomplete epidemiological information, such as prophylaxis, treatment and/or patient compliance with the treatment. It is worth noting that patients in Table 1 reported recurrent symptoms after more than 1 month. This may be attributable to a parasite relapse from latent hypnozoites, since all cases were *P. vivax* and patients did not report additional travels during or after treatment. Also, it is possible that a lack of anti-hypnozoite therapy has increased the probability of these relapses (which could be higher than 20%, according to previous reports) [50].

## Conclusion

It is essential to strengthen the malaria surveillance system in Chile, which should include complete epidemiological data, travel history, previous diagnostic results and prophylaxis or treatment given. This information is valuable to better assist patients, provide recommendations to travellers and further analyse the current malaria situation in the country. Moreover, the introduction of malaria cases in Chile has brought the presence of resistant strains from all over the world, which requires proper control and surveillance.

## Data Availability

The datasets used during the current study are available from the corresponding author on reasonable request, but most of the data generated during this study are included in this published article.

## Abbreviations

ACT: Artemisinin-based combination therapy
CDC: Centers for Disease Control and Prevention
CQ: Chloroquine
ISP: Public Health Institute of Chile
MS: Microsatellites
PET: Photo-induced electron transfer
PQ: Primaquine
PAHO: Pan American Health Organization
SP: Sulfadoxine–Pyrimethamine
WHO: World Health Organization

## References

[1] WHO. Malaria. Geneva: World Health Organization. http://www.who.int/ith/diseases/malaria/en/. Accessed 10 Jan 2019.

[2] Amir A, Cheong FW, de Silva JR, Liew JWK, Lau YL (2018). *Plasmodium knowlesi* malaria: current research perspectives. Infect Drug Resist..

[3] WHO. World malaria report 2018. Geneva: World Health Organization. https://apps.who.int/iris/bitstream/handle/10665/275867/9789241565653-eng.pdf. Accessed 5 Mar 2019.

[4] OPS/OMS. Situación de la Malaria en la Región de las Américas, 2000–2016. Organización Panamericana de la Salud, Organización Mundial de la Salud. https://www.paho.org/hq/index.php?option=com_docman&view=download&category_slug=statistics-data-maps-8109&alias=45344-situation-malaria-region-americas-2000-2016-344&Itemid=270&lang=es. Accessed 10 Jan 2019.

[5] Grillet ME, Villegas L, Oletta JF, Tami A, Conn JE (2018). Malaria in Venezuela requires response. Science.

[6] Recht J, Siqueira AM, Monteiro WM, Herrera SM, Herrera S, Lacerda MVG (2017). Malaria in Brazil, Colombia, Peru and Venezuela: current challenges in malaria control and elimination. Malar J..

[7] OPS/OMS. Actualización Epidemiológica Aumento de malaria en las Américas. Organización Panamericana de la Salud, Organización Mundial de la Salud. https://www.paho.org/hq/index.php?option=com_docman&view=download&category_slug=2018-9582&alias=43437-30-enero-2018-malaria-actualizacion-epidemiologica-437&Itemid=270&lang=es. Accessed 10 Jan 2019.

[8] Schenone H, Olea A, Rojas A, García N (2002). Malaria en Chile: 1913–2001. Rev Med Chile..

[9] González CR, Sallum MA (2010). *Anopheles (Nyssorhynchus) atacamensis* (Diptera: Culicidae), a new species from Northern Chile. Mem Inst Oswaldo Cruz.

[10] WHO. Guidelines for the treatment of malaria, third edition. Geneva: World Health Organization. http://apps.who.int/iris/bitstream/handle/10665/162441/9789241549127_eng.pdf?sequence=1. Accessed 10 Jan 2019.

[11] Noedl H, Se Y, Schaecher K, Smith BL, Socheat D, Fukuda MM (2008). Evidence of artemisinin-resistant malaria in Western Cambodia. N Engl J Med.

[12] Dondorp AM, Nosten F, Yi P, Das D, Phyo AP, Tarning J (2009). Artemisinin resistance in *Plasmodium falciparum* malaria. N Engl J Med.

[13] Takala-Harrison S, Laufer MK (2015). Antimalarial drug resistance in Africa: key lessons for the future. Ann N Y Acad Sci.

[14] Chenet SM, Akinyi Okoth S, Huber CS, Chandrabose J, Lucchi NW, Talundzic E (2016). Independent emergence of the *Plasmodium falciparum* Kelch propeller domain mutant allele C580Y in Guyana. J Infect Dis.

[15] Straimer J, Gnädig NF, Witkowski B, Amaratunga C, Duru V, Ramadani AP (2015). K13-propeller mutations confer artemisinin resistance in *Plasmodium falciparum* clinical isolates. Science.

[16] Fairhurst RM, Dondorp AM (2016). Artemisinin-resistant *Plasmodium falciparum* malaria. Microbiol Spec..

[17] Lucchi NW, Narayanan J, Karell MA, Xayavong M, Kariuki S, DaSilva AJ (2013). Molecular diagnosis of malaria by photo-induced electron transfer fluorogenic primers: PET-PCR. PLoS ONE.

[18] Akerele D, Ljolje D, Talundzic E, Udhayakumar V, Lucchi NW (2017). Molecular diagnosis of *Plasmodium ovale* by photo-induced electron transfer fluorogenic primers: PET-PCR. PLoS ONE.

[19] Vinayak S, Alam T, Mixson-Hayden T, McCollum AM, Sem R, Shah NK (2010). Origin and evolution of sulfadoxine resistant *Plasmodium falciparum*. PLoS Pathog.

[20] Griffing S, Syphard L, Sridaran S, McCollum AM, Mixson-Hayden T, Vinayak S (2010). *pfmdr1* amplification and fixation of *pfcrt* chloroquine resistance alleles in *Plasmodium falciparum* in Venezuela. Antimicrob Agents Chemother.

[21] Anderson TJC, Su XZ, Bockarie M, Lagog M, Day KP (1999). Twelve microsatellite markers for characterization of *Plasmodium falciparum* from finger-prick blood samples. Parasitology.

[22] McCollum AM, Schneider KA, Griffing SM, Zhou Z, Kariuki S, Ter-Kuile F (2012). Differences in selective pressure on *dhps* and *dhfr* drug resistant mutations in western Kenya. Malar J..

[23] INE. Características de la inmigración internacional en Chile, Censo 2017. Santiago: Instituto Nacional de Estadísticas. http://www.censo2017.cl/descargas/inmigracion/181123-documento-migracion.pdf. Accessed 10 Jan 2019.

[24] Mace KE, Arguin PM, Tan KR (2018). Malaria surveillance—United States, 2015. MMWR Surveill Summ..

[25] Weitzel T, Labarca J, Cortes CP, Rosas R, Balcells ME, Perret C (2015). Cluster of imported vivax malaria in travelers returning from Peru. J Travel Med..

[26] Sá JM, Twu O, Hayton K, Reyes S, Fay MP, Ringwald P (2009). Geographic patterns of *Plasmodium falciparum* drug resistance distinguished by differential responses to amodiaquine and chloroquine. Proc Natl Acad Sci USA.

[27] Awasthi G, Satya GBK, Das A (2012). *Pfcrt* haplotypes and the evolutionary history of chloroquine-resistant *Plasmodium falciparum*. Mem Inst Oswaldo Cruz.

[28] Wootton JC, Feng X, Ferdig MT, Cooper RA, Mu J, Baruch DI (2002). Genetic diversity and chloroquine selective sweeps in *Plasmodium falciparum*. Nature.

[29] Zhou RM, Zhang HW, Yang CY, Liu Y, Zhao YL, Li SH (2016). Molecular mutation profile of *pfcrt* in *Plasmodium falciparum* isolates imported from Africa in Henan province. Malar J..

[30] Ndam NT, Basco LK, Ngane VF, Ayouba A, Ngolle EM, Deloron P (2017). Reemergence of chloroquine-sensitive *pfcrt* K76 *Plasmodium falciparum* genotype in southeastern Cameroon. Malar J..

[31] Fançony C, Gamboa D, Sebastião Y, Hallett R, Sutherland C, Sousa-Figueiredo JC (2012). Various *pfcrt* and *pfmdr1* genotypes of *Plasmodium falciparum* cocirculate with *P. malariae*, *P. ovale* spp., and *P. vivax* in Northern Angola. Antimicrob Agents Chemother..

[32] Fançony C, Brito M, Gil JP (2016). *Plasmodium falciparum* drug resistance in Angola. Malar J..

[33] Niang M, Marrama L, Ekala MT, Alioune G, Tall A, Ndiaye JL (2008). Accumulation of CVIET *Pfcrt* allele of *Plasmodium falciparum* in placenta of pregnant women living in an urban area of Dakar, Senegal. J Antimicrob Chemother..

[34] Golassa L, Enweji N, Erko B, Aseffa A, Swedberg G (2014). High prevalence of *pfcrt*-CVIET haplotype in isolates from asymptomatic and symptomatic patients in south-central Oromia, Ethiopia. Malar J..

[35] Sáenz FE, Morton LC, Okoth SA, Valenzuela G, Vera-Arias CA, Vélez-Álvarez E (2015). Clonal population expansion in an outbreak of *Plasmodium falciparum* on the northwest coast of Ecuador. Malar J..

[36] Cui L, Mharakurwa S, Ndiaye D, Rathod PK, Rosenthal PJ (2015). Antimalarial drug resistance: literature review and activities and findings of the ICEMR network. Am J Trop Med Hyg.

[37] Taylor AR, Flegg JA, Holmes CC, Guérin PJ, Sibley CH, Conrad MD (2017). Artemether-lumefantrine and dihydroartemisinin-piperaquine exert inverse selective pressure on *Plasmodium falciparum* drug sensitivity-associated haplotypes in Uganda. Open Forum Infect Dis..

[38] Elbadry MA, Existe A, Victor YS, Memnon G, Fukuda M, Dame JB (2013). Survey of *Plasmodium falciparum* multidrug resistance-1 and chloroquine resistance transporter alleles in Haiti. Malar J..

[39] Veiga MI, Dhingra SK, Henrich PP, Straimer J, Gnädig N, Uhlemann AC (2016). Globally prevalent PfMDR1 mutations modulate *Plasmodium falciparum* susceptibility to artemisinin-based combination therapies. Nat Commun..

[40] Anderson TJC, Haubold B, Williams JT, Estrada-Franco JG, Richardson L, Mollinedo R (2000). Microsatellite markers reveal a spectrum of population structures in the malaria parasite *Plasmodium falciparum*. Mol Biol Evol.

[41] Nderu D, Kimani F, Karanja E, Thiong’o K, Akinyi M, Too E (2019). Genetic diversity and population structure of Plasmodium falciparum in Kenyan-Ugandan border areas. Trop Med Int Health..

[42] Touray AO, Mobegi VA, Wamunyokoli F, Herren JK (2020). Diversity and multiplicity of *P. falciparum* infections among asymptomatic school children in Mbita, Western Kenya. Sci Rep..

[43] Murray L, Mobegi VA, Duffy CW, Assefa SA, Kwiatkowski DP, Laman E (2016). Microsatellite genotyping and genome-wide single nucleotide polymorphism-based indices of *Plasmodium falciparum* diversity within clinical infections. Malar J..

[44] Dorado EJ, Okoth SA, Montenegro LM, Diaz G, Barnwell JW, Udhayakumar V (2016). Genetic characterisation of *Plasmodium falciparum* isolates with deletion of the *pfhrp2* and/or *pfhrp3* genes in Colombia: the Amazon Region, a challenge for malaria diagnosis and control. PLoS ONE..

[45] Vera-Arias CA, Castro LE, Gómez-Obando J, Sáenz FE (2019). Diverse origin of *Plasmodium falciparum* in northwest Ecuador. Malar J..

[46] Abukari Z, Okonu R, Nyarko SB, Lo AC, Dieng CC, Salifu SP (2019). The diversity, multiplicity of infection and population structure of *P. falciparum* parasites circulating in asymptomatic carriers living in high and low malaria transmission settings of Ghana. Genes..

[47] Oyebola MK, Idowu ET, Nyang H, Olukosi YA, Otubanjo OA, Nwakanma DC (2014). Microsatellite markers reveal low levels of population sub-structuring of *Plasmodium falciparum* in southwestern Nigeria. Malar J..

[48] Carter TE, Malloy H, Existe A, Memnon G, St. Victor Y, Okech BA (2015). Genetic diversity of *Plasmodium falciparum* in Haiti: insights from microsatellite markers. PLoS ONE..

[49] MINSAL. Orientaciones técnicas para el diagnóstico y tratamiento de la malaria en Chile Santiago: Ministerio de Salud Pública de Chile. https://diprece.minsal.cl/wrdprss_minsal/wp-content/uploads/2016/06/Orientaciones-t%C3%A9cnicas-para-el-diagn%C3%B3stico-y-tratamiento-de-la-Malaria.pdf. Accessed 10 Jan 2019.

[50] McCollum AM, Soberon V, Salas CJ (2014). Genetic variation and recurrent parasitaemia in Peruvian *Plasmodium vivax* populations. Malar J..

